# Differences in Parotid Dosimetry and Expected Normal Tissue Complication Probabilities in Whole Brain Radiation Plans Covering C1 Versus C2

**DOI:** 10.7759/cureus.1217

**Published:** 2017-05-03

**Authors:** Andrew Orton, John Gordon, Tyler Vigh, Allison Tonkin, George Cannon

**Affiliations:** 1 Radiation Oncology, University of Utah School of Medicine, Huntsman Cancer Institute; 2 Radiation Oncology, Intermountain Medical Center; 3 Radiology, Intermountain Medical Center

**Keywords:** xerostomia, palliation, dosimetry, ntcp, complications

## Abstract

**Objectives:**

There is no consensus standard regarding the placement of the inferior field border in whole brain radiation therapy (WBRT) plans, with most providers choosing to cover the first versus (vs.) second cervical vertebrae (C1 vs. C2). We hypothesize that extending coverage to C2 may increase predicted rates of xerostomia.

**Methods:**

Fifteen patients underwent computed tomography (CT) simulation; two WBRT plans were then produced, one covering C2 and the other covering C1. The plans were otherwise standard, and patients were prescribed doses of 25, 30 and 37.5 gray (Gy). Dose-volume statistics were obtained and normal tissue complication probabilities (NTCPs) were estimated using the Lyman-Burman-Kutcher model. Mean parotid dose and predicted xerostomia rates were compared for plans covering C2 vs. C1 using a two-sided patient-matched t-test. Plans were also evaluated to determine whether extending the lower field border to cover C2 would result in a violation of commonly accepted dosimetric planning constraints.

**Results:**

The mean dose to both parotid glands was significantly higher in WBRT plans covering C2 compared to plans covering C1 for all dose prescriptions (p<0.01). Normal tissue complication probabilities were also significantly higher when covering C2 vs. C1, for all prescribed doses (p<0.01). Predicted median rates of xerostomia ranged from <0.03%-21% for plans covering C2 vs. <0.001%-12% for patients treated with plans covering C1 (p<0.01), dependent on the treatment dose and NTCP model. Plans covering C2 were unable to constrain at least one parotid to <20 Gy in 31% of plans vs. 9% of plans when C1 was covered. A total parotid dose constraint of <25 Gy was violated in 13% of plans covering C2 vs. 0% of plans covering C1.

**Conclusions:**

Coverage of C2 significantly increases the mean parotid dose and predicted NTCPs and results in more frequent violation of commonly accepted dosimetric planning constraints.

## Introduction

Whole brain radiation therapy (WBRT) is one of the most common treatments delivered in radiation oncology. Whole brain radiation therapy is typically utilized in the palliative setting when treating patients with multiple brain metastases. Less frequently, it is delivered in the curative setting, including as prophylactic cranial irradiation (PCI) for small-cell lung cancer and various high-risk or central nervous system (CNS) positive leukemias, as well as part of the definitive regimen in some institutions for primary CNS lymphomas. Several recent papers have proposed that the parotid glands be considered at-risk organs in patients receiving WBRT [1-2]. The poor prognosis traditionally associated with brain metastases previously limited interest in evaluating the long-term side effects of WBRT, as it was not expected that patients would live long enough to experience these late effects. However, the potential late effects of WBRT, including xerostomia, have become more prescient given the improved overall survival achieved in patients with brain metastases [3], as well as the potential for xerostomia to be induced during or shortly after radiation [4]. Additionally, it has been observed that the long-term impact of radiation on salivary function exists as a continuum and that doses as low as 10-15 gray (Gy) may decrease parotid gland function [5]. In their review of publications that related dose-volume statistics to radiotherapy-induced xerostomia, Eisbruch, et al. noted that a lower mean dose to the parotid gland usually results in better salivary function, even at doses <10 Gy [6]. Specifically, they recommended keeping the dose to at least one gland <20 Gy. Another commonly accepted guideline to prevent xerostomia is to keep the mean dose to both parotids below 25 Gy [7]. When the mean dose to both parotids is limited to less than 25 Gy, it has been estimated that salivary flow will return to baseline by 12 months [8]. Because xerostomia can be quite detrimental to the patient’s quality of life, there has been interest in evaluating and subsequently limiting the dose to the parotid glands when delivering WBRT [9-11].

Whole brain radiation therapy is most commonly delivered using opposed lateral fields with 6 MV photons with gantry-rotation or multi-leaf collimators (MLCs) optimized to spare the lenses bilaterally. Even in the computed tomography (CT) era, radiation treatment fields continue to be designed based on anatomic landmarks identifiable on lateral radiographs of the skull, with a 5 to 10 mm margin placed on the cribriform plate and floor of the middle cranial fossa and 1 to 2 cm of “flash” beyond the anterior, superior, and posterior cranium to ensure dose homogeneity [12]. Variations in the WBRT technique can lead to substantial differences in the mean parotid dose [13]. The dose of radiation delivered incidentally to the parotid glands is influenced primarily by the location of the inferior border of the treatment field. Most commonly, the inferior border for the field is placed at either the inferior endplate of the C1 or C2 vertebra. The decision of which vertebral level to cover is typically a matter of physician preference, although coverage through C2 has most commonly been recommended when treating small-cell lung cancer and leukemia via the German Helmet technique [12,14]. The purpose of this study is to compare the dose delivered to the parotid glands in plans covering C1 versus C2. Normal tissue complication probabilities (NTCP) for these two approaches were then calculated and compared.

## Materials and methods

**Figure 1:**
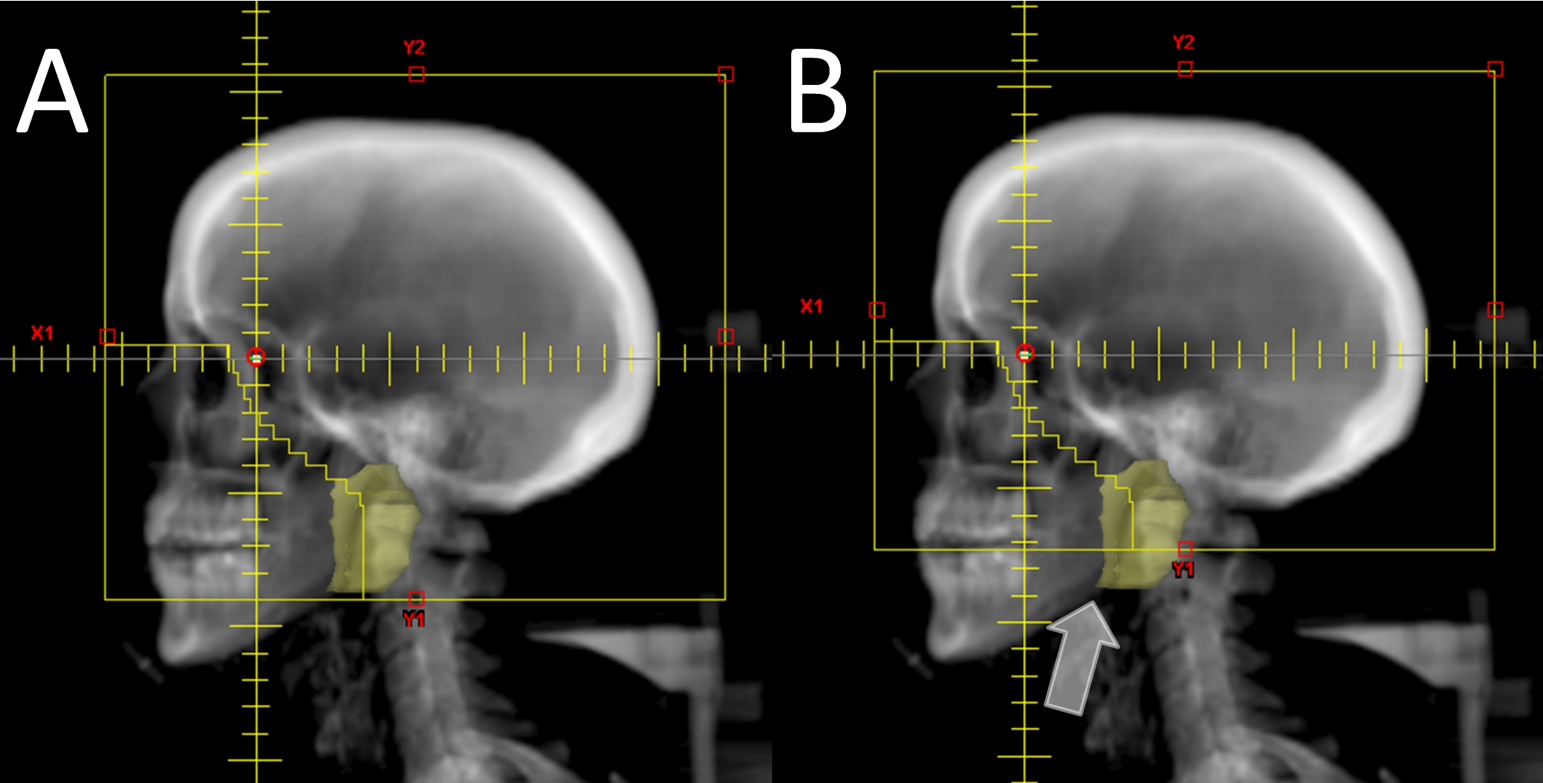
An example of differences in parotid coverage when the lower field is set at C1 vs. C2 An example of a WBRT plan with coverage of C2 (panel A) vs. C1 (panel B). The parotid volume is shown in yellow. The portion of the parotid blocked by the inferior primary jaw of the C1 plan is indicated by the white arrow.

Fifteen patients underwent computed tomography (CT) simulation in our facility following immobilization with thermoplastic masks in the supine position. CT images were obtained from the vertex of the skull to the top of the thoracic spine at 2.5 mm intervals. The right and left parotid glands were contoured by a resident physician (AO) and the contours were subsequently reviewed independently by an attending radiation oncologist (GC) and an attending neuroradiologist (AT), with discrepancies resolved by consensus (see Figure 1).

Two treatment plans were produced for each patient using the CT simulation images. In each plan, treatment was delivered using 6 MV photons in an opposed lateral configuration with the first plan extending the inferior field border to include all of C1, while the second plan set the inferior border to include all of C2. The WBRT plans were otherwise standard, with MLCs used to create a 1 cm field edge margin on the bony landmarks demarcating the inferior border of the cranial contents, and isocenters placed within the posterior orbit to create a quasi-half beam technique and limit divergence to the contralateral lens. Given the variations in the PCI and WBRT doses employed, plans were generated for prescription doses of 25 Gy, 30 Gy and 37.5 Gy.

Dose-volume statistics were obtained using the Varian Eclipse External Beam Planning System. The normal tissue complication probability (NTCP) was estimated in the same manner published by Noh et al.; the Lyman-Burman-Kutcher model was used with parameters obtained from studies published by Eisbruch [6], Emami [15] and Roesink [16]. Mean dose to the parotids and NTCPs were compared between the two groups, and statistical significance was determined using a patient-matched two-sided t-test. Plans were also evaluated to determine whether extending the lower field border to cover C2 would result in the violation of commonly accepted dosimetric planning constraints (limiting at least one parotid to a mean dose of <20 Gy, and limiting both parotids to <25 Gy).

## Results

**Figure 2:**
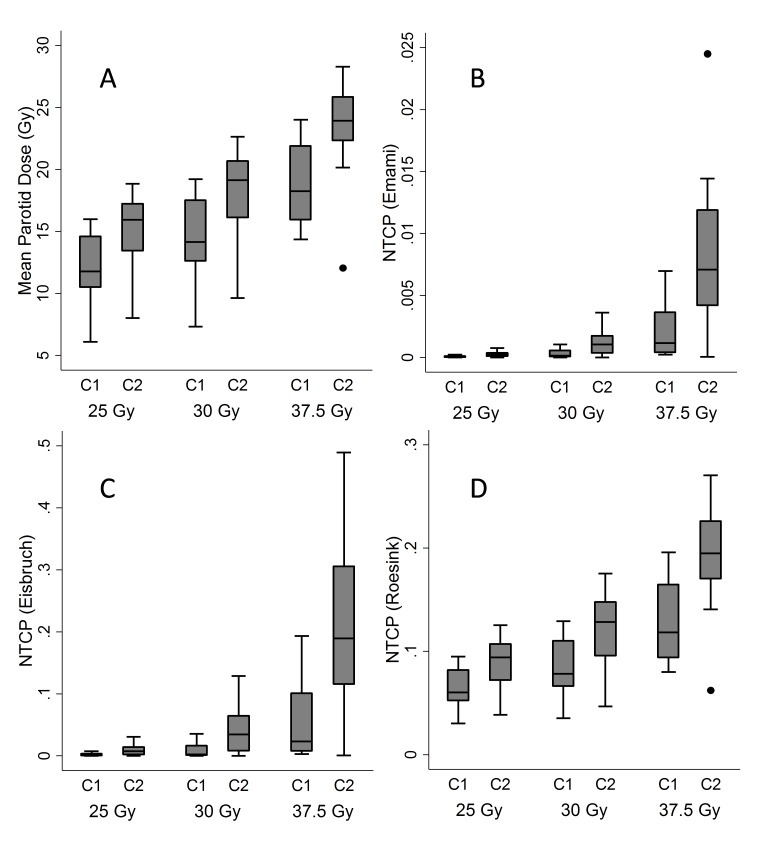
A comparison of expected xerostomia rates Box and whisker plots representing the distribution of mean parotid dose (panel A), and NTCPs derived from parameters proposed by Emami, et al. (panel B), Eisbruch, et al. (panel C), and Roesink (panel D). Horizontal lines represent mean values for all 15 test patients, while the boxes span the 25th percentile to 75th percentile. Hashes denote 95th percentiles and dots represent observations falling outside of the box and whisker distribution.

The mean dose to both parotid glands was significantly higher in WBRT plans covering C2 compared to plans limited to covering C1 for 25 Gy, 30 Gy and 37.5 Gy plans (see Figure 2, panel A). Dosimetric differences between C1 plans and C2 plans are given in Table 1. The mean parotid dose was 15.3 Gy vs. 11.9 Gy (p<0.01) for the 25 Gy plan; 18.3 Gy vs. 14.3 Gy (p<0.01) for the 30 Gy plan; and 23.4 Gy vs. 18.5 Gy (p<0.01) for the 37.5 Gy plan. These trends were unchanged when evaluating the right and left parotid glands individually or when combined as a single dosimetric volume.

**Table 1:** Differences in mean parotid dose, predicted NTCP rates, and dosiometric planning violations between plans covering C2 vs. C1 NTCP: Normal tissue complication probabilities; V10: Volume of tissue receiving 20 Gy; V25: Volume of tissue receiving 25 Gy.

	25 Gy		p	30 Gy		37.5 Gy		
	C2	C1		C2	C1	C2	C1	p
Mean parotid dose (Gy)	15.3	11.9	<0.01	18.3	14.3	23.4	18.5	<0.01
V20	47.7%	33.1%	<0.01	54.9%	39.0%	61.2%	45.5%	<0.01
V25	6.8%	3.0%	<0.01	45.3%	31.3%	56.2%	40.9%	<0.01
NTCP (Emami)	0.03%	0.007%	<0.01	0.1%	0.03%	1.5%	0.2%	<0.01
NTCP (Eisbruch)	1.0%	0.2%	<0.01	4.2%	0.8%	21.1%	4.9%	<0.01
NTCP (Roesink)	9.1%	6.3%	<0.01	12.3%	8.2%	19.2%	12.5%	<0.01
Violations of planning constraints								
Total parotid dose <25 Gy	0/15	0/15		0/15	0/15	6/15	0/15	
At least one gland <20 Gy	0/15	0/15		3/15	0/15	10/15	4/15	

Normal tissue complication probabilities for the parotid gland were also significantly higher when covering C2 vs. C1, for all prescribed doses (see Figure 2, panels B, C, and D). Mean predicted NTCPs for plans covering C2 vs. C1 are summarized in Table 1. Predicted rates of xerostomia, calculated using parameters proposed by Roesink et al. [16], ranged from 9%-19% vs. 6%-12% for patients treated with plans covering C2 vs. C1 (p<0.001). Estimates generated using the parameters suggested by Eisbruch, et al. [6] ranged from 1%-21% vs. 0.1%-5% in plans covering C2 vs. C1. Using the parameters suggested by Emami, et al. [15], we estimated the xerostomia risk to be very low at <0.03%-1% vs. >0.001%-0.2% for plans covering C2 vs. C1.

The effect of covering C2 vs. C1 on meeting commonly accepted planning constraints is summarized in Table 1. In our series, when the prescribed dose was 25 or 30 Gy, we found that all patient plans limited to C1 were able to constrain at least one parotid to a mean dose <20 Gy. In contrast, 3/15 (20%) plans covering to C2 did not meet this criterion when 30 Gy was prescribed to cover C2. When the prescription dose was increased to 37.5 Gy, 4/15 (27%) of C1 plans and 10/15 (67%) of C2 plans exceeded the constraint. Plans covering C1 and plans covering C2 were able to meet the total parotid dose constraint of 25 Gy when the dose prescription was 25 or 30 Gy. When the prescription dose was increased to 37.5 Gy, all plans covering C1 met this constraint, but 6/15 plans covering C2 did not.

## Discussion

Xerostomia secondary to radiation-induced impairment of salivary flow can be a significant quality-of-life (QOL) issue for patients, resulting in dysphagia, dysgeusia, and increased risk of dental infections and caries. Salivary dysfunction is of particular concern in both the PCI and palliative WBRT settings. For patients cured following PCI for small-cell lung cancer and various high-risk or CNS-positive leukemias, chronic xerostomia can impact long-term QOL. The prospect of worsened QOL from xerostomia is also of special concern in the palliative setting as treatment toxicities must necessarily be balanced with the dual goals of life prolongation and maintenance of QOL.

The parotid glands are the dominant producers of serous saliva and are at risk for significant incidental radiation dose when delivering WBRT [1,2,7,17]. While historical data had suggested a salivary gland tolerance dose of ~40 Gy [18], it is clear that parotid gland tolerance is in fact much lower than this. Eisbruch and colleagues reported in a seminal publication that a mean parotid dose of 26 Gy was associated with the threshold for impairment of stimulated salivary flow [6]. In this report, most glands that received over 26 Gy produced little saliva with no significant recovery through time. Partial volume thresholds were also reported by these investigators. Since the publication of this report, numerous other reports have characterized the dose-response relationship [18-19]. Results have varied regarding the predicted dose that would lead to xerostomia, but xerostomia with mean doses as low as 22 Gy has been identified [20].

Although data indicate recovery of salivary gland function with time, it can take up to 12 months to recover to pretreatment levels even when mean doses to the parotids are kept below 25 Gy [18]. Quantitative Analyses of Normal Tissue Effects in the Clinic (QUANTEC) guidelines therefore recommend sparing one parotid gland to a mean dose <20 Gy if the paired gland is to receive a high dose or, if that is not possible, to limit the mean total bilateral parotid dose to <25 Gy [5,19].

The radiation technique used to deliver WBRT can significantly affect the mean dose to the parotid glands due to their anatomic location [10,13]. There is historic concern that tightening the inferior borders of standard WBRT fields may result in underdosing the brain parenchyma [20-22]. Dosimetric studies have shown that in the 3-D planning era, the additional information provided by CT scans can limit dose to normal tissue while maintaining appropriate coverage of the intracranial space [11-13,23-25].

There is no set standard for the lower border of the WBRT field. Coverage to the bottom of the C1 or C2 vertebra originated in an era prior to cross-sectional imaging, with treatment to such osseous anatomic landmarks adhered to in order to avoid marginal tumor misses. The variable coverage of C1 vs. C2 seems to have been dictated by institutional preferences and practices rather than clinical data. Unfortunately, radiation-induced xerostomia is likely underreported in the palliative and prophylactic setting, leaving clinicians to depend on NTCP models to guide treatment planning decisions. We feel that the principle of primum non nocere should guide physicians to set the border at C1 unless radiographic or clinical disease characteristics indicate a high chance of failure at the inferior margin.

## Conclusions

Extending the inferior border of a standard whole brain field to cover the C2 vertebral body significantly increases the mean parotid dose and the resultant anticipated rates of clinical xerostomia based on three separate NTCP models. Whole brain radiation therapy plans that cover C2 more often result in the violation of commonly accepted parotid treatment planning constraints compared to plans which are limited to covering C1. Clinicians who choose to prescribe dose to cover C2 in WBRT plans should be aware that doing so significantly increases the dose of radiation delivered to the parotid glands, and is predicted to result in more incidence of xerostomia at rates that may be as high as 20% in plans delivering 37.5 Gy. Given the rarity of failures within the craniospinal axis at the C2 level, we would advocate against coverage of C2 in WBRT.
